# Prevalence of *Toxoplasma* Infection in Mexican Newborns and Children: A Systematic Review from 1954 to 2009

**DOI:** 10.5402/2012/501216

**Published:** 2012-09-25

**Authors:** Ma. de la Luz Galvan-Ramírez, Rogelio Troyo-Sanroman, Sonia Roman, Rosamaría Bernal-Redondo, José Luís Vázquez Castellanos

**Affiliations:** ^1^Neurophysiology Laboratory, Departament of Physiology, Health Science University Center, University of Guadalajara, Sierra Mojada No. 950 Edificio N, Col. Independencia, Guadalajara, México 44320 JAL, Mexico; ^2^Department of Molecular Biology in Medicine, Civil Hospital of Guadalajara “Fray Antonio Alcalde”, University of Guadalajara, Hospital 278, 44280 Guadalajara, JAL, Mexico; ^3^“Federico Gómez”, Hospital Infantil de México, Secretaria de Salud, Mexico; ^4^Pediatric and Epidemiology Service, Hospital Number 110 Mexican Institute of Social Security, Avenue Circunvalación Oblatos and Francisco Bocanegra, Guadalajara, JAL, Mexico

## Abstract

*Introduction*. Recent studies in Mexico have shown that from 20/10,000 to 58/10,000 newborns with *Toxoplasma* infection could be undetected. The aim of this study was to determine the weighed prevalence of *T. gondii* infection and describe the epidemiological transition of infection in newborns. *Methods*. Research literature reporting *Toxoplasma* infection prevalence in Mexican newborns and children were searched in five international databases. Weighted prevalence was calculated by inverse variance-weighted method in asymptomatic and symptomatic study groups, and the epidemiological transition was estimated by a lineal regression analysis. *Results*. The weighed prevalence in 4833 asymptomatic newborns was 0.616%, CI95% (0.396%–0.835%) (*P* < 0.001), whereas, among 895 symptomatic newborns, the weighed prevalence was 3.02%, CI 95% (1.91%–4.1%) (*P* < 0.001). A downward trend of 0.25%/year represented an accumulated decrease of −13,75% in the prevalence in the symptomatic newborns throughout 55 years, whereas, in the asymptomatic children, the prevalence was similar over the course of the years. *Conclusion*. The high-weighted prevalence of congenital *Toxoplasma* infection in newborns justifies that *Toxoplasma gondii* testing be included in the screening programs for women during pregnancy and newborns in Mexico. A rapid diagnosis and treatment strategy could aid in limiting a potential damage to the newborns.

## 1. Introduction

*Toxoplasma gondii* is a coccidian protozoa of the Apicomplexa family that was first described in 1908. It is the causal agent of congenital toxoplasmosis (CT) that occurs when mothers are infected with this protozoan for the first time during pregnancy and by transplacental transmission infecting the fetus [1]. Damage to the visual and central nervous system can be severe if the fetus is infected during the first trimester [2]; during the second and third trimesters, disorders are less severe and newborns can present an asymptomatic infection and later on develop chorioretinitis, mental retardation, and sensory damage [3]. It can also occur at final term, during placental detachment, or while in delivery. 

At least, 15 distinct *T. gondii* strain types have been found throughout the world by enzyme-linked immunoabsorbent assay (ELISA) [1]. Strain types II and not exclusively II (NE-II) have been detected by specific antibodies that recognize allelic peptide motifs of the distinct strain types. NE-II strain is associated with premature birth and infants infected with severe manifestations of disease than infants infected by strain type II parasites [4].

CT is a health problem in Mexico. Although it was first reported in 1954, CT infection in newborns has been poorly studied in Mexico. Two studies using ELISA filter paper have been performed; one prospective study in 2005 reported a congenital infection rate of two in 1,000 live births in Mexico City [5]. In the state of Jalisco (west Mexico) five out of 860 live births had *Toxoplasma* infection [6]. In the same state, a prevalence of 2.72% was reported in a cohort of 807 pediatric patients that were clinically monitored for infectious diseases and ophthalmological sequelae during three years [7]. 

Several diagnostic assays for *T. gondii *infection that differ in sensitivity and specificity have been used in Mexico over time. The Sabin-Feldman dye test (SF) is the golden standard by which other methods such as complement fixation (CF), indirect immune fluorescence (IFAT), enzyme-linked immunoabsorbent assay-filter paper (ELISA-FP) are evaluated. 

The variability in the diagnostic methods and the low amount of cases included in some studies led us to apply a mathematical method to analyze the epidemiological data of *T. gondii* infection in newborns and congenital toxoplasmosis in Mexico. Therefore, the aim of this study was to determine the frequency of infection by *T. gondii* and to describe its epidemiological transition from 1954 to 2009 among Mexican newborns. 

## 2. Material and Methods 

### 2.1. Data Collection

An electronic literature search was performed with a time span set from December of 1954 to December of 2009 in the following databases: Pubmed, Google Scholar, and Latindex. The keyword combinations were the following: *Toxoplasma *infection in newborns, congenital toxoplasmosis, epidemiology of infection in newborns, prevalence of toxoplasmosis in Mexico, anti-*Toxoplasma* antibodies alone or combined with different diagnostic methods: Sabin-Feldman dye test (SF), complement fixation (CF), indirect immune fluorescence (IFAT), enzyme-linked immunoabsorbent assay-filter paper (ELISA-FP). 

### 2.2. Eligibility Criteria

We decided to include in the meta-analysis only those studies that met the following criteria. (1) Full-text publications that included data such as year of publication, characteristics of the study population, location, sample size, number of positive cases, and diagnostic test. Abstracts from scientific meetings were also collected and considered as potentially relevant.

### 2.3. Data Extraction

Data of interest was categorized into two types of study groups: (1) asymptomatic newborns and (2) symptomatic newborns and children. Asymptomatic newborns did not present symptoms of toxoplasmosis at birth although some did develop clinical manifestations afterwards. Symptomatic newborns were born with clinical manifestations of toxoplasmosis.

### 2.4. Statistical Methods

#### 2.4.1. Crude Prevalence (CP)

Crude prevalence was estimated as the number of positive cases divided by the sample size of the cohort in each study group.

#### 2.4.2. Population Prevalence (Weighed Prevalence (WP))

The procedure to calculate the population prevalence of *T. gondii* in all groups or subgroups included in this meta-analysis was based on the formula *P* = ∑(*pi*)(1/*vi*)/∑1/*vi* as explained by Borenstein et al. [8]. 

#### 2.4.3. Definitions

*i* is number of studies in each group, *Ni* is total number of cases in each study, *Ai* is number of positive cases from each study, and (*Ni* − *Ai*) is the number of negative cases in each study. The risk of infection as a proportion in each study (*pi*) was calculated as *Ai*/*Ni*. The variance of each study (*v*_*i*_) was calculated as *Ai*(*Ni* − *Ai*)/*Ni* ^3^. The standard error (SE_*i*_) of each study was estimated as $\sqrt{vi}$. 

The total population variance (*V*) was estimated as 1/∑1/*vi*. The standard error of the population was calculated as $\text{SE}=\sqrt{V}$. The confidence interval (C.I.95%) for the population prevalence was obtained by *P* + 1.96 SE (upper limit) and *P* − 1.96 EE lower limit. The probability that the prevalence value could be different from zero was calculated with a *Z* test, *Z* = *P*/*SE*. 

#### 2.4.4. Lineal Regression Analysis

Bivariate simple linear regression analysis (time in years, from 1954 versus crude prevalence) was conducted to determine the relationship between the seroprevalence of *Toxoplasma* infection over time. The regression coefficient was calculated by the linear equation (*y* = *a* + *bx*), *a* is ordinate at the origin, *b* is slope, and the value of *R* ^2^ and *p* was obtained with SPSS program. The epidemiological behavior of the prevalence of *Toxoplasma* infection was estimated by plotting the year of each publication date (independent variable) starting at year 1954 until 2009 versus the corresponding prevalence (dependent variable) reported in each study [8].

## 3. Results

A total of eight publications met the eligibility criteria for meta-analysis that included 10 studies; one paper included three groups representing 5728 newborns and children with clinical toxoplasmosis. Seven studies were performed in healthy newborns and three studies in infants with congenital infection.

### 3.1. Asymptomatic Newborns

A total of 4,833 newborns were reported in five publications. The first two studies were performed by the SF method that included 2,186 and 329 newborns from Mexico City, respectively [9, 10]. A third study was carried out in 367 live births by using the CF method [11]. Three more studies, one performed in Mexico City and two in Jalisco, included 1001, 860, 60 cases of apparently healthy newborns, and 30 cases of premature newborns. In this last study, mother newborns and IgM-positive cases were confirmed in both. Newborns were asymptomatic to congenital toxoplasmosis, but were referred to the neurology and ophthalmology services to be evaluated and observed in a two-year followup [5, 6, 12]. The meta-analysis of the healthy newborns group revealed a weighed prevalence of 0.616%, a population variance of 0.0001%, a standard error of 0.111%, (CI_95%_ 0.396%–0.835%), and a *Z* value =5.5 (*P* < 0.001) (Tables 1 and 2).

### 3.2. Symptomatic Toxoplasmosis in Newborns and Children

A total of three studies including 895 cases were analyzed. The first study included 58 patients from Mexico City diagnosed by the CF technique [13]. In the state of Jalisco, two studies were conducted. In one study, 22 out of 807 children were seropositive for IgM *T. gondii* antibodies and clinical manifestations; seven with ocular lesions, six cerebral and once pulmonary calcifications, seven presented neurological symptoms [7]. In another study, 30 newborns had congenital malformations: 13 were premature and lymphoid disorders, six had hydrocephalus, six had macrocephaly, and four were obits, and one case had hydrothorax [12]. In this last study, mother-newborns were evaluated, and IgM-positive cases were confirmed in both. Newborns were referred to the neurology and ophthalmology services for treatment and control. In these newborns, the weighed prevalence was 3.02%, the population variance was 0.0032%, standard error of 0.5697% (CI_95%_ 1.914%–4.1%), *Z* value =5.331 (*P* < 0.001) (Tables 1 and 2).

### 3.3. Epidemiological Transition of *Toxoplasma* Infection in Mexico

**Figure 1:**
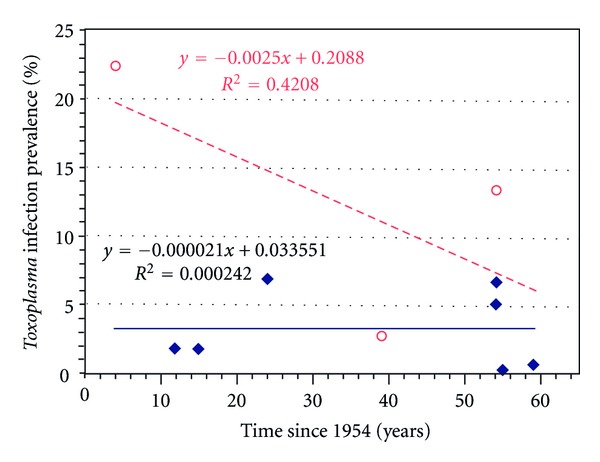
Epidemiological transition of the *T. gondii* infection from 1954 to 2009. (——) linear regression and (♦) prevalence of *Toxoplasma* infection in asymptomatic newborns groups. The downward trend in the prevalence rate was 0.002%/year, (∘) prevalence and (- - -) linear regression in congenital toxoplasmosis groups. The downward trend in the prevalence rate was 0.25%/year. In both groups, the *R* ^2^ value was not statistically significant (NS).

The lineal regression analysis of *Toxoplasma* infection in Mexican children over a time span of 55 years shows a downward trend of 0.25%/year that represents a 13.75% decrease of congenital infection in the symptomatic group, but, in asymptomatic children, the crude prevalence (0.62%) was similar over the course of the years. However, this difference was not significant (Figure 1).

## 4. Discussion

In the healthy newborns that included seven studies with 4833 cases, a weighed prevalence of 0.616% (range: 0.396% to 0.835%) was estimated by meta-analysis. In two postmortem studies: one conducted among 4,197 autopsies of apparently healthy children at the Children's Hospital at Mexico City in 1976 and another performed among 4,000 autopsies in the Pediatric Hospital, the positivity for *Toxoplasma* in tissues was 0.11% and 0.6%, respectively [14, 15]. These results found by Villegas Villegas-González et al. [15] are similar to those found in Jalisco where 5 over 860 infants (0.69%) were positive for *T. gondii* [6]. 

In the group of children with congenital infection, the weighted prevalence was higher (3.02%) than the former group as expected, since these cases had clinical symptoms of CT. 

The epidemiological transition of prevalence of CT throughout 55 years showed a downward trend of 0.25%/year with an accumulated prevalence of 13.75%. Even though it was not significant, this data is significant because it may reflect the effect of the introduction into the country of the ELISA-FP in last 20 years, which is more sensitive and specific than the earlier methods used in the first studies that began 55 years ago.

The epidemiological transition of prevalence during 55 years in asymptomatic live birth was 0.002%/year, and the accumulated tendency was 0.11% (Figure 1). However, the prevalence observed in newborns from México City, 2 in 1000 or (20/10,000) and the prevalence in Jalisco with 5 over 860 or (58/10,000), both are higher than reported in the city of Belém, state of Paraná in Northern Brazil [16]. This prevalence is considered high in comparison to those observed in other countries where neonatal screening programs for CT have been implemented. For instance, in Poland the prevalence rate in newborns is 4.7/10,000, in Sweden 0.73/10,000, in Italy 1.38/10,000, and Denmark 1.6/10,000. Toxoplasmosis serology has been shown to be a valuable tool in neonatal screening programs to prevent serious sequelae and estimate the prevalence of congenital infections [16]. The high prevalence of CT observed throughout Mexico justifies that *Toxoplasma* infection testing should be included in the neonatal screening programs for metabolic disorders. 

The diagnosis of* Toxoplasma* infection in pregnant woman is based by serological methods that detect antibodies. However, Mexico does not have a national program to investigate *Toxoplasma* infection during pregnancy in any health institution, in spite of the high prevalence of IgM anti-*Toxoplasma *antibodies (20.7%) reported in high-risk pregnant women [17]. Moreover, even though the first test of *Toxoplasma* antibodies may be negative, it is particularly convenient to carry out monthly tests in order to monitor seroconversion and to lower the risk of transmission. Both a rapid diagnosis and treatment strategy could aid in limiting a potential damage to the newborns [18, 19].

In Mexico, nearly 2,000,000 children are born each year, so that 4000 and 11,600 congenital infections could be undetected. This study shows that congenital *Toxoplasma *infection is a public health problem in Mexico, which has been dismissed by the national health sector and the low interest of researchers in this area. Therefore, more studies are required together with the implementation of a nationwide detection program in pregnant women as well as monitoring of *Toxoplasma* infection in newborns.

### 4.1. Implications for Research

A crucial factor is the difference in the prevalence of *T. gondii* infections due to the sensitivity and specificity of diagnostic tests, since there are several methods to identify and evaluate antibodies in individuals who were infected by the parasite. At least four different diagnostic assays have been used in this study, that range from the lowest specificity and sensitivity like the CF progressing on to the SF dye test and other similar tests up to the improved ELISA.

## Figures and Tables

**Table 1:** Serological studies in live birth and children with *Toxoplasma* infection.

Ref.	Year	Author	Location	Test	Cases	Positives cases	Crude prevalence (%)	Population
[13]	1954	Gutiérrez et al.	Mexico	CF	58	13	22.4	Symptomatic
[9]	1962	Roch and Bravo Becherelle	Mexico	SF	2,186	40	1.9	Asymptomatic
[10]	1965	Espinosa de los Reyes et al.	Mexico	SF	329	6	1.8	Asymptomatic
[11]	1974	Biagi et al.	Mexico	CF	367	25	6.8	Asymptomatic
[7]	1989	Galván-Ramírez and Garzón de la Mora	Guadalajara	IFAT	807	22	2.7	Symptomatic
[12]	2007	Galván-Ramírez et al.	Guadalajara	IFAT	30	4	13.3	Symptomatic
			Guadalajara	IFAT	30	2	6.6	Asymptomatic
			Guadalajara	IFAT	60	3	5	Asymptomatic
[5]	2005	Vela-Amieva et al.	Mexico	ELISA	1,001	2	0.2	Asymptomatic
[6]	2009	Galvan-Ramirez et al.	Guadalajara	ELISA	860	5	0.6	Asymptomatic

					5728	122		

Ref: reference; CF: complement fixation; SF: Sabin & Feldman dye test, ELISA: enzyme-linked immunosorbent assay; IFAT: immunofluorescence. Reference [12] included three groups.

**Table 2:** Prevalence calculated by meta-analysis in Asymptomatic newborns and Symptomatic congenital *Toxoplasma* infection.

Population	*N*	Number of cases	Positive cases	Crude prevalence (%)	Weighed prevalence (%)	Lower-upper limit (%)
Symptomatic	3	895	39	4.36	3.02	1.9–4.1
Asymptomatic	7	4833	83	1.72	0.61	0.39–0.835

Total	10	5728	122	2.136	3.63	
